# The role of coagulation/fibrinolysis during *Streptococcus pyogenes* infection

**DOI:** 10.3389/fcimb.2014.00128

**Published:** 2014-09-11

**Authors:** Torsten G. Loof, Christin Deicke, Eva Medina

**Affiliations:** Infection Immunology Research Group, Helmholtz Centre for Infection ResearchBraunschweig, Germany

**Keywords:** *Streptococcus pyogenes*, coagulation system, contact system, fibrinogen, fibrinolysis, plasminogen

## Abstract

The hemostatic system comprises platelet aggregation, coagulation and fibrinolysis and is a host defense mechanism that protects the integrity of the vascular system after tissue injury. During bacterial infections, the coagulation system cooperates with the inflammatory system to eliminate the invading pathogens. However, pathogenic bacteria have frequently evolved mechanisms to exploit the hemostatic system components for their own benefit. *Streptococcus pyogenes*, also known as Group A Streptococcus, provides a remarkable example of the extraordinary capacity of pathogens to exploit the host hemostatic system to support microbial survival and dissemination. The coagulation cascade comprises the contact system (also known as the intrinsic pathway) and the tissue factor pathway (also known as the extrinsic pathway), both leading to fibrin formation. During the early phase of *S. pyogenes* infection, the activation of the contact system eventually leads to bacterial entrapment within a fibrin clot, where *S. pyogenes* is immobilized and killed. However, entrapped *S. pyogenes* can circumvent the antimicrobial effect of the clot by sequestering host plasminogen on the bacterial cell surface that, after conversion into its active proteolytic form, plasmin, degrades the fibrin network and facilitates the liberation of *S. pyogenes* from the clot. Furthermore, the surface-localized fibrinolytic activity also cleaves a variety of extracellular matrix proteins, thereby enabling *S. pyogenes* to migrate across barriers and disseminate within the host. This review summarizes the knowledge gained during the last two decades on the role of coagulation/fibrinolysis in host defense against *S. pyogenes* as well as the strategies developed by this pathogen to evade and exploit these host mechanisms for its own benefit.

## Activation of the contact system by *S. pyogenes*

**Figure 1:**
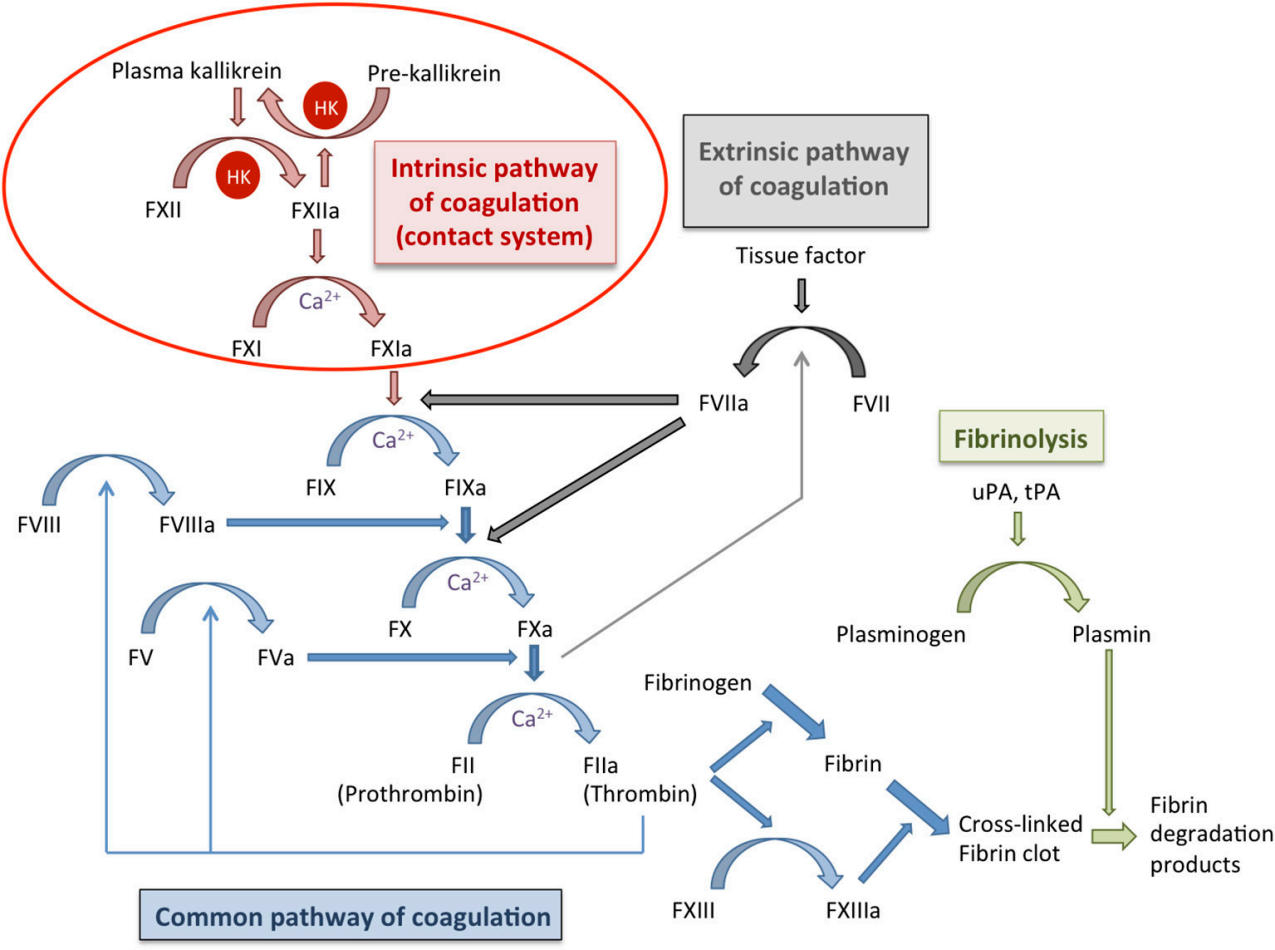
**Schematic representation of the coagulation cascade and the fibrinolytic system**. The coagulation cascade (blue arrows) can be activated during hemostasis via the intrinsic pathway (contact system; red arrows) or the extrinsic pathway (gray arrows) that ultimately converge on the common pathway of coagulation. Both pathways lead to the activation of factor X and subsequently of thrombin, which is required for the conversion of fibrinogen into fibrin and for activation of factor XIII. The fibrin clot is cross-linked and stabilized by factor XIII. Fibrinolysis (green arrows) is activated at the same time that the coagulation system but operates more slowly and is important for the regulation of hemostasis. During fibrinolysis, plasminogen is converted into plasmin that degrades the fibrin network. Coagulation factors are indicated with “F” followed by a roman numeral, an additional “a” denotes the activated form; HK, high molecular weight kininogen; uPA, urokinase plasminogen activator; tPA, tissue plasminogen activator.

The intrinsic pathway of coagulation, also known as kallikrein/kinin system or contact system consists of four components; the serine proteinases factor XI (FXI) and factor XII (FXII), the plasma kallikrein (PK), and the non-enzymatic cofactor, high molecular weight kininogen (HK) (Colman and Schmaier, 1997) (Figure 1). Under physiological conditions, these factors circulate as zymogens in the bloodstream or are assembled on the surface of various cell types including endothelial cells, platelets, and polymorphonuclear neutrophils (Colman and Schmaier, 1997). Despite intensive research, the function of the contact system remains enigmatic. A major role of the contact system in the initiation of the coagulation pathway has been questioned based on the absence of bleeding disorders in individuals with hereditary deficiency on FXII (Zeerleder et al., 1999). This is further supported by the normal bleeding phenotype exhibited by FXII-deficient mice (Renné et al., 2005). Interestingly, FXII deficiency in mice was associated with a profound defect in the formation and stabilization of platelet-rich thrombi (Renné et al., 2005). Thus, it was proposed that activation of the extrinsic pathway of coagulation in response to vascular injury would initiate the thrombus formation, thereby providing a surface for assembly of the contact system that would further support thrombus development and stabilization (Renné et al., 2005, 2012). A role of the contact system in immunity and inflammation has also been suggested (Frick et al., 2007; Renné, 2012).

**Figure 2:**
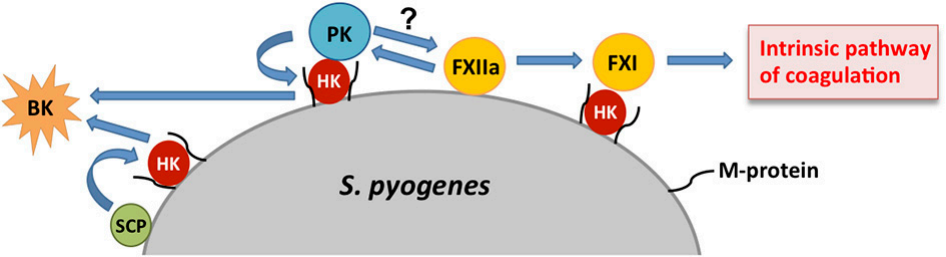
**Schematic representation of contact activation on the surface of *S. pyogenes***. Assembly of coagulation factors on the surface of *S. pyogenes* leads to an autoactivation of factor XII (FXIIa) that, in turn, activates factor XI (FXI) and plasma kallikrein (PK), both anchored to the bacterial surface via high molecular weight kininogen (HK) bound to the streptococcal M protein. Activated FXI triggers the intrinsic pathway of coagulation while PK induces the cleavage of HK resulting in the generation of bradykinin (BK). HK immobilized on the bacterial surface can be also cleaved by the streptococcal cysteine proteinase (SCP).

Activation of the contact system is triggered upon binding of FXII to negatively charged surfaces, such as dextran sulfate or kaolin as well as to several biological activators including DNA, RNA, and collagen (reviewed by Maas et al., 2011). Contact factors can also become activated after binding to bacterial surfaces (reviewed by Nickel and Renné, 2012). Thus, contact factors have been reported to bind to the surface of gram-negative (Herwald et al., 1998; Holm et al., 2011; Murphy et al., 2011; Rapala-Kozik et al., 2011) as well as gram-positive (Ben Nasr et al., 1996, 1997) bacteria. In the particular case of *S. pyogenes*, FXII undergoes autoactivation (FXIIa) after binding to the bacterial surface. Subsequently, FXIIa activates PK and FXI, also anchored to the bacterial surface via HK bound to the streptococcal M protein (Ben Nasr et al., 1995) (Figure 2). Activation of FXI triggers the intrinsic pathway of coagulation leading to the formation of thrombin and of a fibrin clot (Figure 2). On the other hand, activation of PK by FXIIa induces the cleavage of HK and the generation of the vasoactive and proinflammatory peptide bradykinin (BK) (Figure 2). Furthermore, the streptococcal cysteine proteinase SCP has been also identified as one of the bacterial factors involved in the cleavage of HK without requiring previous activation of PK (Herwald et al., 1996) (Figure 2). Based on these observations, it was proposed that the recruitment of high amounts of HK by the M protein on the surface of *S. pyogenes* could lead to a local burst of kinins by the action of SCP. This would result in increased vascular permeability leading to exudation of high amounts of plasma proteins and nutrients into the site of infection (Herwald et al., 1996). Extravasation of massive fluid from the intravascular compartment out into the tissues can induce a drop in blood pressure that can contribute to the development of sepsis and septic shock, which are the most severe clinical outcomes of *S. pyogenes* infections (Henningham et al., 2012). Further evidence supporting an activation of the contact system during invasive streptococcal infection was provided by the prolonged activated partial thromboplastin time (aPTT) observed in mice experimentally infected with *S. pyogenes* (Sriskandan et al., 2000). Prolonged aPTTs have also been detected in patients with septic shock (Smith-Erichsen et al., 1982) and extensive pulmonary hemorrhage has been observed in post-mortem examination of lungs isolated from patients that succumbed after fulminant *S. pyogenes* infection (Ooe et al., 1999).

*S. pyogenes* M1 protein as well as other bacterial virulence factors have been reported to stimulate the production of pro-coagulant microversicles, which are small particles released from the cell membrane of activated or apoptotic cells (Piccin et al., 2007) and from peripheral blood mononuclear cells (Oehmcke et al., 2012). *S. pyogenes* has been shown to induce apoptosis in several host cell types such as neutrophils, keratinocytes and epithelial cells through different virulence factors and this has been suggested to play a role in the infectious process (for review see Ulett and Adderson, 2006). Activation of the contact system on these microvesicles was shown to contribute to clot stabilization and stimulation of inflammatory responses by the generation of BK (Oehmcke et al., 2012). Pro-coagulant monocyte-derived microvesicles were also found in plasma from septic patients (Oehmcke et al., 2012).

In summary, experimental animal studies and clinical data indicate a link between activation of the contact system and invasive *S. pyogenes* infections. Therefore, interfering with the activation of the contact system has been considered as therapeutic intervention to improve the outcome of severe invasive *S. pyogenes* infection. As proof of principle, treatment with a peptide (HKH20) derived from the region of human HK involved in the binding to negative charged surfaces (Hasan et al., 1995), was shown to block the assembly and activation of the contact system and to prevent lung bleedings and early mortality of *S. pyogenes-*infected mice when administered in combination with clindamycin (Oehmcke et al., 2009a).

## The role of the coagulation system in innate host defense against *S. pyogenes*

It has been proposed that the vertebrate coagulation system is evolutionarily a by-product of the innate immune system, with the blood clotting factors having diverged from the complement system (Krem and Di Cera, 2002). In this regard, there is a growing body of evidence suggesting the contribution of the coagulation system to host defense against invading pathogens. The role of the coagulation system in early innate immune defenses is most prominent in *S. pyogenes* infections. For example, the D3 domain of HK, has been reported to exert direct antimicrobial activity against *S. pyogenes* (Frick et al., 2006). HK is organized into six structural domains: domains 1–3 (D1–D3) are related to cystatin, D4 contains the BK peptide, D5 mediates binding to negatively charged surfaces, and the carboxyl-terminal D6 contains a zymogen binding sequence for FXI and prekallikrein which, with D5, accounts for its cofactor activity (Colman and Schmaier, 1997). Although intact HK does not have anti-bacterial effect, the D3 fragments released after enzymatic cleavage of HK by for example neutrophils elastase (Vogel et al., 1988), possess strong bactericidal activity (Frick et al., 2006). Thus, it was proposed that, after assembly and activation of the contact system on bacterial surfaces, HK can be cleaved by elastase released by activated neutrophils, resulting in the simultaneous release of antibacterial D3 fragments and BK that will further promote inflammation and plasma leakage (Frick et al., 2006). Other components of the contact system such as the domain D5 of HK have been also reported to exert antimicrobial effect (Nordahl et al., 2005).

**Figure 3:**
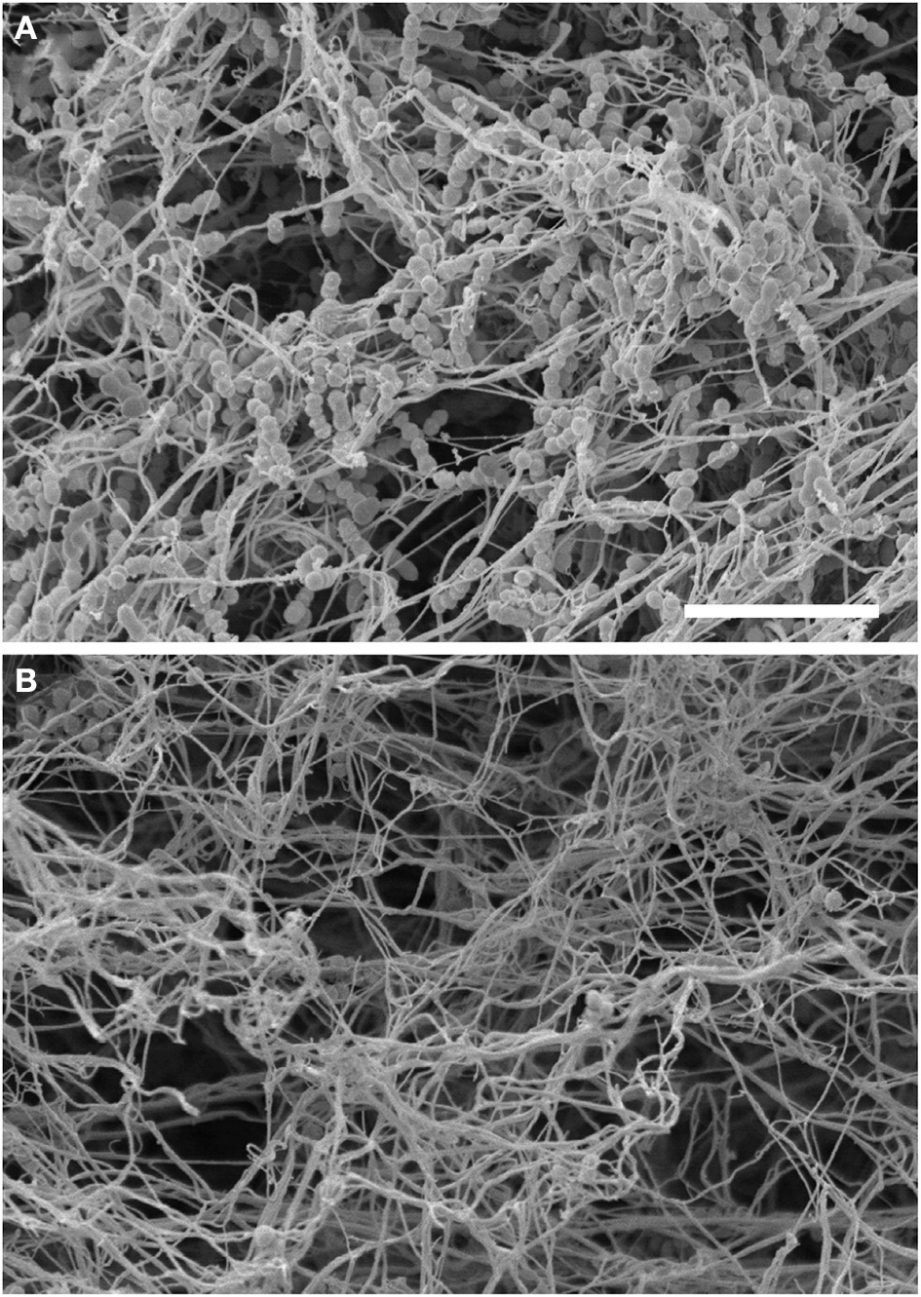
**Entrapment of *S. pyogenes* within a fibrin clot is mediated by Factor XIII**. Scanning electron micrograph of *S. pyogenes* bacteria of strain AP1 (M1 serotype) within a clot generated in normal human plasma **(A)** or in Factor XIII-deficient human plasma **(B)**. The scale bar represents 10 μm.

Contact activation leads to the induction of the entire coagulation cascade and there is evidence that entrapment of microorganisms after clot formation may hamper their dissemination from the local site of infection. This mechanism appears to be highly conserved during evolution from invertebrates to humans (Rotstein, 1992; Sun et al., 2004; Matsuda et al., 2007; Loof et al., 2011a) and it has been designated as “immunothrombosis” by Engelmann and Massberg (2013). The ultimate goal of the coagulation pathway is to produce thrombin, which is generated from its inactive precursor prothrombin by the prothrombinase complex formed by FXa and its co-factor FVa. The main functions of thrombin are the conversion of fibrinogen to fibrin as well as the activation of coagulation FXIII. Fibrin strands are then cross-linked by activated FXIII (FXIIIa) to form a stable blood clot (Figure 1). During the process of thrombin generation in blood, part of the early-generated thrombin feeds back on the cascade system to activate FV and FVIII (Figure 1), thus promoting further thrombin generation (Brummel et al., 2002). Thrombin generation takes place on the surface of activated platelets that form the primary hemostatic plug (Brass, 2003). The relevance of thrombin generation in antimicrobial host defense against *S. pyogenes* was examined by Sun and co-workers using mice with deficiency of FV in either the plasma or the platelet compartment (Sun et al., 2009). They reported that reduction of FV in either pool resulted in increased mortality of mice after subcutaneous inoculation of *S. pyogenes*, thus supporting a role of thrombin generation in host defense against this pathogen (Sun et al., 2009). The authors hypothesized that local fibrin deposition mediated by both the platelet and plasma FV pools could reduce the survival and dissemination of *S. pyogenes* by limiting vascular invasion and hematogenous spread (Sun et al., 2009). The capacity of the fibrin clot to immobilize and kill *S. pyogenes* was first demonstrated by Shannon et al. (2010). They also demonstrated that histidine-rich glycoprotein (HRP), an abundant plasma protein, played a critical role in this defense mechanism (Shannon et al., 2010). Thus, clots formed in HRG-deficient plasma were less effective than those formed in normal plasma at entrapping and killing *S. pyogenes* (Shannon et al., 2010). Furthermore, HRG-deficient mice exhibited increased susceptibility to *S. pyogenes* infection than wild-type mice, a phenotype that was reverted after supplementing the HRG-deficient mice with purified HRG (Shannon et al., 2010). FXIII was also shown to be an important factor for the entrapment of different bacterial pathogens within clots since clots generated in plasma from FXIII-deficient individuals lost the capacity to immobilize bacteria (Wang et al., 2010). Therefore, FXIII-mediated entrapment of pathogens within a fibrin clot seems to be a general immune defense mechanism against bacterial pathogens (Wang et al., 2010). It has also been reported that FXIII mediates cross-linking of bacterial surface structures, such as the M1 protein of *S. pyogenes*, to fibrin fibers within the clot, where the microorganisms are subsequently killed by the induction of plasma-derived antimicrobial activity (Loof et al., 2011b). The electron microscopy photographs depicted in Figure 3 show the FXIII-mediated entrapment of *S. pyogenes* bacteria of strain AP1 (serotype M1) in a fibrin clot. A large amount of bacteria were enwoven within the fibrin network when the clot was generated in normal human plasma (Figure 3A) whereas only a few bacteria are found loosely associated with the clot when FXIII-deficient plasma was used (Figure 3B). It was also reported that mice deficient in FXIII exhibited higher levels of local inflammation than wild-type mice after subcutaneous inoculation with *S. pyogenes* (Loof et al., 2011b). Furthermore, streptococci were found confined within the fibrin meshwork in skin biopsies from infected wild-type mice, but they were spread throughout the site of infection in infected FXIII-deficient mice (Loof et al., 2011b). A similar immobilization of bacteria within fibrin networks was also observed in skin biopsies obtained from patients with severe *S. pyogenes* infections (Loof et al., 2011b). These observations underscore the relevance of FXIII in the early control of *S. pyogenes* infection.

## The role of fibrinogen in innate host defense against *S. pyogenes*

Fibrinogen is a plasma glycoprotein synthesized in the liver that is essential for blood coagulation. In order to form a clot, soluble fibrinogen must be converted into insoluble fibrin polymer by thrombin (Blombäck et al., 1978). Fibrinogen has been suggested to play an important role in host defense against *S. pyogenes* by limiting both bacterial hematogenous dissemination and by promoting inflammation leading to bacterial clearance (Sun et al., 2004, 2009). Thus, it was reported that reduction of fibrinogen levels after injection of snake venom enhanced the mortality of *S. pyogenes-*infected mice (Sun et al., 2004). Furthermore, mice deficient in the production of fibrinogen (Fga^−/−^) were also highly susceptible to *S. pyogenes* (Sun et al., 2009).

The important role played by fibrinogen for confining and neutralizing *S. pyogenes* during the early infection was further demonstrated by Påhlman et al. (2013). This group showed that the peptide fragment GHR28 released from the β-chain of fibrinogen after activation with thrombin exerted antimicrobial activity against *S. pyogenes* organisms entrapped within a fibrin clot (Påhlman et al., 2013). Nevertheless, binding of plasminogen to the bacterial surface was a pre-requisite for effective bacterial killing (Påhlman et al., 2013). Kantor and Cole described for the first time the capacity of a *S. pyogenes* factor, which they named FPF (fibrinogen precipitating factor), to precipitate human and bovine fibrinogen (Kantor and Cole, 1959). In further experiments, Kantor confirmed the identity of FPF and M protein (Kantor, 1965). The M protein is the major virulence determinant of *S. pyogenes* by virtue of its ability to promote the survival of the bacterium in human blood (Fischetti, 1989; Oehmcke et al., 2010). M protein is exposed on the surface of *S. pyogenes* but it can also be released after proteolytic cleavage by cysteine proteinases also secreted by *S. pyogenes* (Berge and Björck, 1995). Due to its high affinity to bind fibrinogen, the release of high amounts of M protein during severe *S. pyogenes* infection can have grave consequences for the patient. In this regard, it was demonstrated that rapid formation of M1 protein/fibrinogen complexes in plasma-activated neutrophils resulted in high levels of plasma leakage, which is one of the events involved in the development of life-threatening streptococcal septic shock (Herwald et al., 2004). To clarify the molecular mechanism underlying the activation of neutrophils by M1 protein/fibrinogen complexes, Macheboeuf and colleagues determined the resolution crystal structure of an M1 protein/fibrinogen complex (Macheboeuf et al., 2011). They found that a conformationally dynamic coiled-coil dimer of M1 organized four fibrinogen molecules into a specific cross-like pattern that supported the construction of a supramolecular network (Macheboeuf et al., 2011). This supramolecular structure presented high densities of integrin-binding sites required for neutrophil activation (Macheboeuf et al., 2011). The M1 protein/fibrinogen supramolecular networks contributed to pathogenesis since blocking M1-fibrinogen interactions was shown to reduce *S. pyogenes* virulence both *in vitro* and *in vivo* (Uchiyama et al., 2013). Fibrinogen binding to surface-exposed M protein was also reported to confer bacterial resistance to phagocytosis by inhibiting complement deposition via the classical pathway (Carlsson et al., 2005). However, the functional effect of M protein-fibrinogen interactions differs depending on the M protein type. Thus, whereas binding of fibrinogen by the M5 protein inhibited complement activation (Carlsson et al., 2005), the binding of fibrinogen to M6 protein had not effect on complement deposition or resistance to phagocytosis (Horstmann et al., 1992). The reasons for these differences remain a matter of debate.

Fibrinogen has been also reported to serve as a docking molecule that attaches *S. pyogenes* to the pro-coagulant microvesicles described in a previous section (Oehmcke et al., 2013). This interaction leads to an alteration of the bacterial surface into a pro-coagulative state and immobilization of *S. pyogenes* within a clot (Oehmcke et al., 2013). The clots generated in the presence of pro-coagulant microvesicles have antimicrobial activity against *S. pyogenes*, which could be mediated by the antimicrobial peptides and other immune response proteins present in the microvesicles (Oehmcke et al., 2013). Local treatment of *S. pyogenes*-infected mice with pro-coagulant microvesicles results in reduced bacterial spreading and improved survival of infected animals indicating that interaction of *S. pyogenes* with pro-coagulant microvesicles may be an integral part of the innate immune response to this pathogen (Oehmcke et al., 2013). Oehmcke and colleagues also reported that fibrinogen in complex with M1 protein can trigger polymorphonuclear neutrophils to release extracellular traps (NETs) (Oehmcke et al., 2009b), which are made of processed chromatin bound to granular and selected cytoplasmic proteins (Brinkmann et al., 2004). Therefore, they proposed that the recruitment and activation of the contact system by NETs amplifies the innate immune response during streptococcal infection (Oehmcke et al., 2013).

## Exploitation of host fibrinolytic system by *S. pyogenes*

Fibrinolysis is the physiological process by which insoluble fibrin clots are removed through enzymatic digestion of the cross-linked fibrin polymers (Cesarman-Maus and Hajjar, 2005). Under physiological conditions, both coagulation and fibrinolysis are strictly regulated to assure blood fluidity while preventing blood loss (Kolev and Machovich, 2003). Plasmin is the major fibrinolytic protease responsible for the lysis of fibrin to produce smaller fragments resulting in the degradation of fibrin clots. Furthermore, plasmin is capable of degrading extracellular matrix proteins and activates matrix metalloproteinases leading to tissue damage (Plow et al., 1995; Monea et al., 2002). Plasmin is generated from circulating plasma plasminogen by both tissue plasminogen activator (tPA) as well as by urokinase (uPA) (Figure 1). These activators are present at various anatomical sites such as the vascular endothelium.

**Figure 4:**
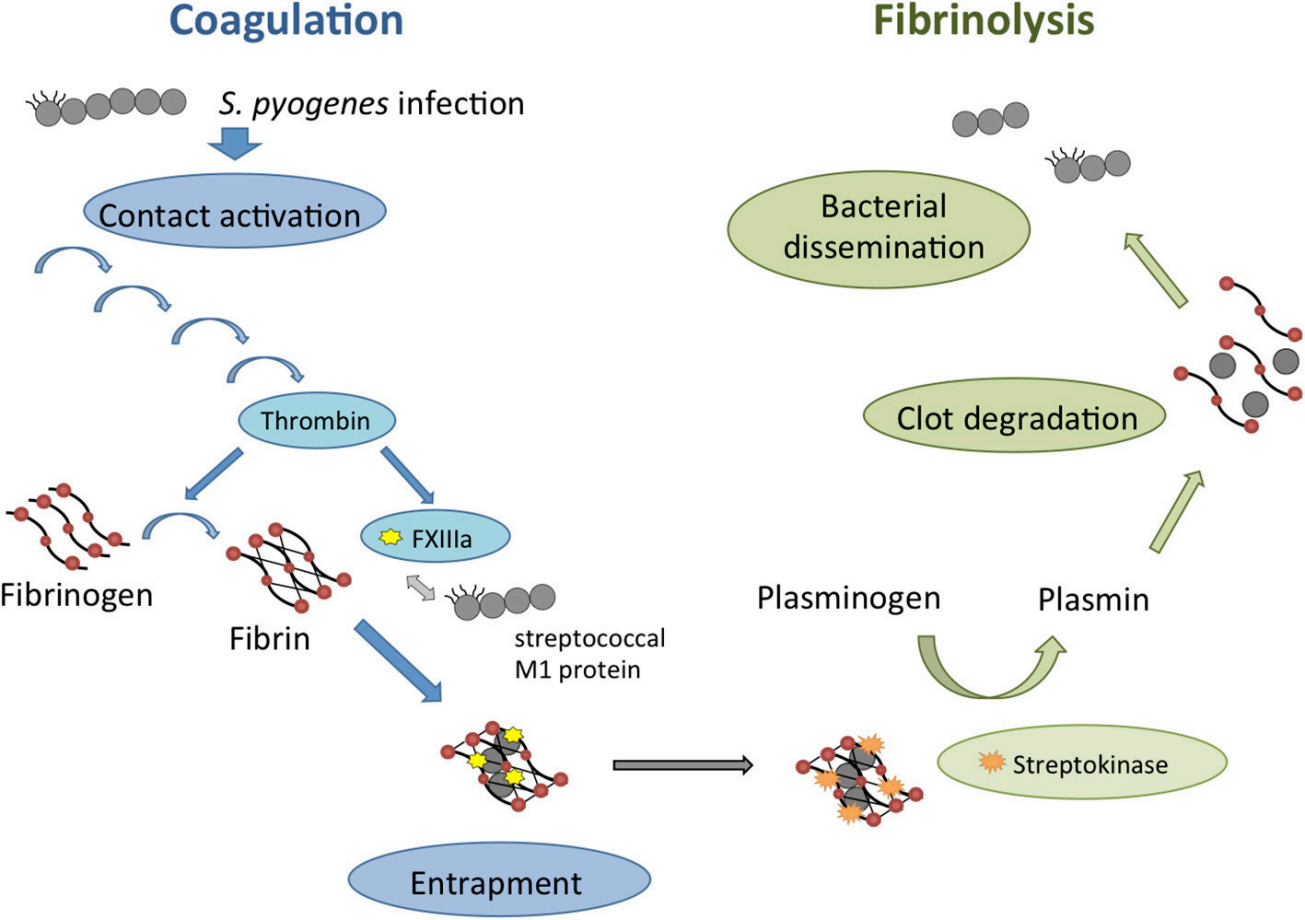
**Schematic overview of the interactions between *S. pyogenes* and the coagulation/fibrinolytic system during infection**. Contact system activation upon *S. pyogenes* infection is leading to the induction of the entire coagulation cascade (blue arrows). Thrombin-activated factor XIII (FXIIIa) targets surface structures of *S. pyogenes* such as the M1 protein and leads to bacterial entrapment within the fibrin network of the clot. *S. pyogenes* can counteract this mechanism by inducing fibrinolysis (green arrows) through the secretion of Streptokinase. Fibrinolysis is leading to clot degradation and therefore enables the dissemination of *S. pyogenes*.

As described above, activation of the coagulation system upon infection can lead to entrapment of *S. pyogenes* within the fibrin network, thus preventing bacterial spreading. To counteract this entrapment, *S. pyogenes* has evolved strategies to induce fibrinolysis and to escape from the clot as depictured in Figure 4. The fibrinolytic activity of *S. pyogenes* was described already in 1933 (Tillett and Garner, 1933). Garner and Tillett identified the streptococcal factor responsible for the fibrinolytic activity that they designated as Fibrinolysin (Garner and Tillett, 1934) and later was renamed Streptokinase (SK) (Green, 1948). SK produced by *S. pyogenes* is highly specific for human plasminogen (Marcum and Kline, 1983). To overcome this limitation and evaluated the role of SK in experimental murine infection models, Khil et al. (2003) co-administered exogenous human plasminogen together with *S. pyogenes* into mice. They reported enhanced skin lesions areas and greater mortality in mice inoculated with wild-type *S. pyogenes* co-administered with human plasminogen that in mice inoculated with SK-deficient *S. pyogenes* also in the presence of human plasminogen (Khil et al., 2003). Similarly, enhanced virulence of *S. pyogenes* was observed by Li et al. (1999) after pre-incubating streptococci in human plasma but not in plasminogen-depleted plasma. The important role of plasminogen in the pathogenesis of *S. pyogenes* infection was further demonstrated using “humanized” transgenic mice expressing human plasminogen (Sun et al., 2004). The transgenic mice expressing human plasminogen exhibited greater susceptible to subcutaneously inoculated *S. pyogenes* than mice expressing the native murine plasminogen (Sun et al., 2004). Furthermore, the increased susceptibility of human plasminogen transgenic mice to *S. pyogenes* was largely abrogated after deletion of SK (Sun et al., 2004). Thus, the SK-plasminogen interaction was demonstrated to be an important determinant of *S. pyogenes* invasiveness *in vivo*. These authors (Sun et al., 2004) also examined the impact on bacterial pathogenicity of recruiting plasminogen by protein PAM, which is expressed by a subset of *S. pyogenes* strains associated with skin infections (Bessen et al., 1996). PAM-expressing streptococci acquired surface-bound plasmin that contributed to bacterial virulence (Ringdahl et al., 1998). Human plasminogen transgenic mice were much more susceptible to PAM-expressing *S. pyogenes* than to an isogenic derivative deficient in the expression of PAM, even in the presence of SK (Sun et al., 2004). Therefore, the ability to recruit plasminogen at the bacterial surface provides and additional mechanism of *S. pyogenes* to promote pericellular fibrinolysis and degradation of components of extracellular matrix and subsequent tissue invasion. Based on these observations, pharmacological disruption of SK activity has been proposed as potential therapeutic strategy to treat severe invasive *S. pyogenes* infections (McArthur et al., 2012). In this regard, a compound designated as CCG-2979, was identified as capable to inhibit gene expression of SK through a high-throughput, growth-based screen on a library of 55,000 small molecules (Sun et al., 2012). Treatment with CCG-2979 enhanced both granulocyte phagocytosis and killing of *S. pyogenes in vitro* and protected mice from *S. pyogenes*-induced mortality (Sun et al., 2012). A series of novel compounds analogs to CCG-2979 has been constructed by Yestrepsky et al. (2013) with improved physico-chemical properties and potent *Staphylococcus aureus* biofilm inhibitory effect. The anti-infective efficacy of these new compounds during *in vivo* infection remains to be determined.

Surface-acquired plasmin activity by *S. pyogenes* has also been shown to degrade host cathelicidin antimicrobial peptides (Hollands et al., 2012). Cathelicidin peptides are important for host defense against *S. pyogenes* (Nizet et al., 2001). Therefore, the co-optation of the host protease activity represents an additional mechanism of *S. pyogenes* to resist the antimicrobial effect of cathelicidins.

## Concluding remarks

The experimental evidence discussed in this review clearly demonstrates a bi-directional relationship between the coagulation/fibrinolytic system and *S. pyogenes*. A schematic overview of these interactions is depictured in Figure 4. Coagulation is important in host responses to *S. pyogenes* and the activation of the contact system and local formation of fibrin prevents spread of the invading pathogen. However, *S. pyogenes* is also capable to hijack the fibrinolytic system to evade this host response. *S. pyogenes* exploits the host fibrinolysis not only for dismantling the imprisoning fibrin clot but also for degrading the extracellular matrix and invade the surrounding tissue. Hence, the balance between the antimicrobial effect of the coagulation system and the bacterial exploitation of the host fibrinolysis is a critical point that will determine the progression of *S. pyogenes* infection. Therefore, pharmacological intervention that will balance coagulation and fibrinolysis may be of benefit for the treatment of severe *S. pyogenes* infection.

### Conflict of interest statement

The authors declare that the research was conducted in the absence of any commercial or financial relationships that could be construed as a potential conflict of interest.
